# Combined Use of Febuxostat and Colchicine Does Not Increase Acute Hepatotoxicity in Patients with Gout: A Retrospective Study

**DOI:** 10.3390/jcm9051488

**Published:** 2020-05-15

**Authors:** Yoon-Jeong Oh, Ki Won Moon

**Affiliations:** Division of Rheumatology, Department of Internal Medicine, Kangwon National University School of Medicine, Chuncheon 24289, Korea; yjgark640@gmail.com

**Keywords:** gout, febuxostat, colchicine, hepatotoxicity, prophylaxis

## Abstract

Colchicine has been effectively used to prevent acute flares in patients with gout, but drug-related adverse events have frequently occurred. We investigated whether colchicine therapy with febuxostat is associated with hepatotoxicity in gout patients. Gout patients treated with (*n* = 121) or without (*n* = 57) colchicine were enrolled upon initiating febuxostat as a urate-lowering treatment, and clinical and laboratory data at diagnosis were compared. Logistic regression analysis was performed to evaluate the risk factors related to hepatotoxicity. Median age of the with-colchicine and without-colchicine groups was 51.0 (37.0–62.0) and 56.0 (43.5–68.5) years, respectively. During the three months of febuxostat prescription, the prevalence of hepatotoxicity was 13/121 (10.9%) in the with-colchicine group and 4/57 (7.0%) in the without-colchicine group, without statistical significance. The rate of colchicine use was not different between the study subjects with or without hepatotoxicity (76.5% vs. 67.1%, *p* = 0.587). Pre-existing liver disease was significantly associated with increased risk of hepatotoxicity after febuxostat treatment (odds ratio, 4.083; 95% confidence interval, 1.326–12.577; *p* = 0.014). Colchicine may be safely used as a prophylactic agent for gout patients with febuxostat. However, upon initiating febuxostat, it is recommended to monitor the development of acute liver injury in gout patients with underlying liver disease.

## 1. Introduction

Gout is a common and treatable form of inflammatory arthritis resulting from the chronic deposition of monosodium urate crystals, which form in the presence of increased urate concentrations [1]. Recent studies have reported that incidence and prevalence rates of gout are rapidly increasing in many countries due to various factors, such as change of dietary habits and comorbid conditions [2,3]. Previous studies have also reported that gout is associated with a number of comorbidities, including cardiovascular disease (CVD), type II diabetes, obesity, dyslipidemia, chronic kidney disease (CKD), nonalcoholic fatty liver disease, and metabolic syndrome [4]. These comorbidities play an important role in determining the medication for treatment options in patients with gout.

Early episodes of acute gouty attack resolve spontaneously within several days or weeks, but repeated acute flares can lead to chronic arthritis with the formation of tophi and joint damage, which contribute to disability and decreased quality of life. Therefore, uric acid-lowering therapy (ULT) as well as prophylaxis of acute attack is one of the treatment goals of gout [5]. A recent guideline for gout management has recommended that when initiating ULT, prophylactic treatment with anti-inflammatory drugs for at least 6 months reduces the frequency of gout flares [6].

Colchicine is a systemic anti-inflammatory agent, and has been regarded as a first line prophylactic drug to prevent gout flare. However, it also has many side effects, such as gastrointestinal symptoms (including diarrhea), muscle pain or weakness, drug-to-drug interactions, renal impairment, and abnormal liver function tests [7]. Therefore, before colchicine treatment, it is necessary to consider the underlying diseases and concomitant medications.

A previous study has shown that colchicine is associated with a risk of hepatotoxicity in gout patients prescribed febuxostat [8], which has also been reported to induce acute liver injury [9]. However, there are few studies regarding hepatic safety of colchicine as a prophylactic therapy in gout patients treated with febuxostat. We investigated whether the concomitant use of colchicine and febuxostat increases hepatotoxicity in gout patients, and evaluated the factors associated with hepatotoxicity in gout patients treated with febuxostat.

## 2. Materials and Methods

### 2.1. Study Subjects

A total of 319 patients initially diagnosed with gout at Kangwon National University Hospital from January 2012 to December 2018 were included. Exclusion criteria were as follows: age at the time of diagnosis <18 years, patients who used uric acid-lowering agents in asymptomatic hyperuricemia, and patients whose follow-up period was less than 3 months. Patients who had a history of allopurinol use were also excluded. A total of 178 gout patients treated with febuxostat were included. The study was approved by the Institutional Review Board of Kangwon National University Hospital and conducted in accordance with the Declaration of Helsinki (IRB protocol number: 2019-12-009).

### 2.2. Data Collection

All data were retrieved from electronic medical records of Kangwon National University Hospital. Demographic data, including age, gender, concomitant medications (uric acid-lowering agents, colchicine, aspirin, diuretics including furosemide, and thiazide), and comorbidities data (hypertension, diabetes mellitus, CVD, heart failure, dyslipidemia, liver cirrhosis, fatty liver, CKD, and dementia), were collected. Liver disease (as defined as liver cirrhosis or fatty liver) was diagnosed by abdominal ultrasound or abdominal computed tomography. We also collected the following biochemical laboratory data: uric acid, aspartate aminotransferase (AST), alanine aminotransferase (ALT), blood urea nitrogen (BUN), creatinine (Cr), total cholesterol, triglyceride, low-density lipoprotein (LDL) and high-density lipoprotein (HDL), at time of diagnosis. In addition, uric acid, AST, ALT, BUN, and Cr were obtained one and three months after initiating febuxostat.

### 2.3. Definition of Hepatotoxicity

Hepatotoxicity was defined as more than three times the upper normal limit when the baseline AST/ALT was normal, and double the baseline AST/ALT when the baseline was abnormally elevated [10].

### 2.4. Statistical Analysis

Continuous variables were expressed as the mean ± standard deviation (SD) or as the median (interquartile range, IQR), while categorical variables were expressed as number percentages (%). The Chi-square test was used to compare the categorical data between the colchicine users and nonusers. Continuous values were compared using the Student’s t-test for parametric data or the Mann–Whitney U test for nonparametric data. Multivariate logistic regression analysis was performed to estimate the relative risk of hepatotoxicity in the study subjects. Age, dosage of febuxostat, ALT, hyperlipidemia, and liver disease identified by univariate analysis as significant predictors of hepatotoxicity (with a *p*-value < 0.2) were included in the multivariate model. Subgroup analysis was also performed; patients with liver cirrhosis were excluded. All statistical analyses were performed using SPSS (version 23.0, Chicago, IL, USA). A *p*-value less than 0.05 was considered statistically significant.

## 3. Results

### 3.1. Baseline Characteristics of Gout Patients with or without Colchicine

**Figure 1:**
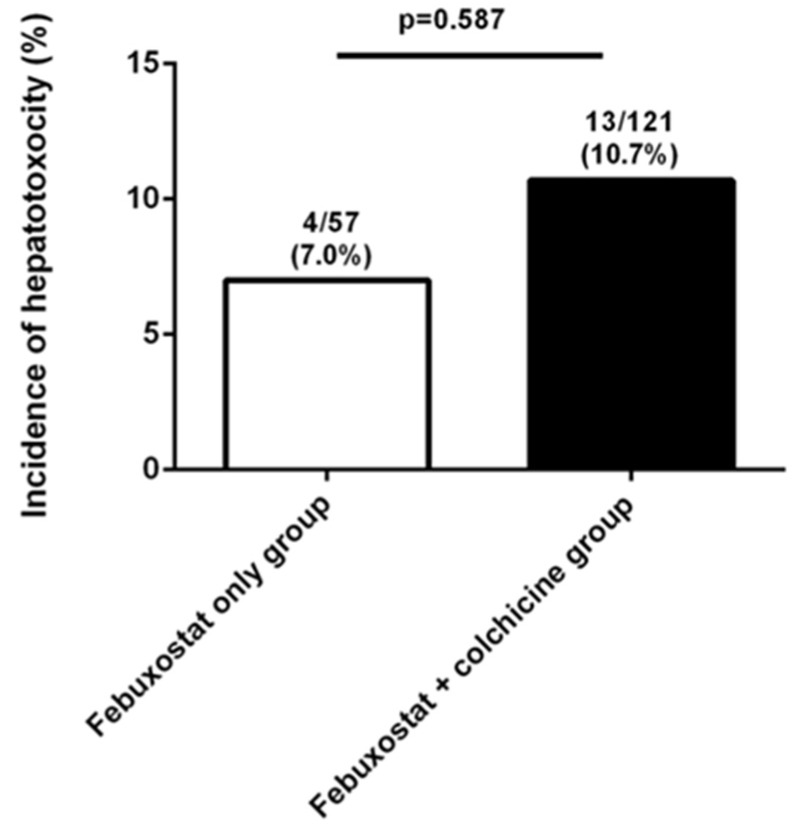
Incidence of hepatotoxicity between the groups with or without colchicine in patients with gout treated febuxostat.

The baseline characteristics of the study patients (*n* = 178) with or without prophylactic colchicine are shown in Table 1. Of the 178 patients, 121 (69.7%) used prophylactic colchicine with febuxostat. The median age (IQR) of colchicine users was 51.0 (37.0–62.0) years, and those without colchicine was 56.0 (43.5–68.5) years, which was not significantly different. The two groups did not differ in terms of disease duration, symptom duration, duration of febuxostat use, dosage of febuxostat, baseline laboratory findings (including uric acid, AST, ALT, and lipid profile), and comorbidities (CVD, dyslipidemia, liver disease, and dementia). There was no difference in the hepatotoxicity between the febuxostat with and without colchicine groups (13/121 [10.7%] vs. 4/57 [7.0%], *p* = 0.587) (Figure 1). Subgroup analysis according to diabetes or CVD revealed no statistically significant differences in the development of hepatotoxicity between the patients with and without colchicine.

However, the laboratory results indicating renal function were significantly worse in patients without colchicine than those with colchicine. In addition, the use of colchicine was significantly less in patients with hypertension, diabetes mellitus, heart failure, and CKD. When initiating ULT, gout flares occurred more frequently in patients without colchicine than those with colchicine (47.1% [24/51] vs. 12.4% [14/113], *p* < 0.001). Diuretics were more frequently used in patients without colchicine than those with colchicine (26.3% vs. 6.6%, *p* = 0.001). Among the 37 patients with liver disease, 30 were diagnosed with alcoholic or nonalcoholic fatty liver and seven were diagnosed with liver cirrhosis. No patients presented with viral hepatitis.

### 3.2. Comparison of Baseline Characteristics According to Hepatotoxicity in Gout Patients on Febuxostat

**Figure 2:**
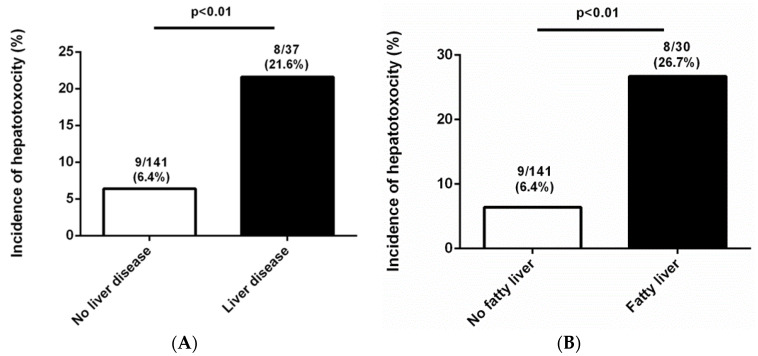
(**A**) Incidence of hepatotoxicity between the groups with or without liver disease in patients with gout treated febuxostat. (**B**) Incidence of hepatotoxicity between the groups with or without fatty liver in patients with gout treated febuxostat.

Among the 178 patients, 17 subjects (9.6%) developed hepatotoxicity within three months after initiating febuxostat treatment. The baseline characteristics of gout patients with or without hepatotoxicity are shown in Table 2. The two groups did not differ in age, sex, disease duration, symptom duration, duration of febuxostat use, dosage of febuxostat or colchicine, and use of concomitant medications (aspirin or diuretics). The rate of colchicine use was not different between the groups with or without hepatotoxicity. In addition, the two groups did not differ in comorbidities except for liver disease. Strikingly, only pre-existing liver disease was significantly higher in patients with hepatotoxicity than in those without hepatotoxicity (8 [47.1%] vs. 29 [18%], *p* = 0.01). Incidence of hepatotoxicity was significantly more frequent in study subjects with liver disease than those without liver disease (Figure 2A). With the exception of cirrhotic patients, the incidence of hepatotoxicity was also high in patients with a fatty liver (Figure 2B). Baseline laboratory parameters, including uric acid, AST, and ALT, were similar between the two groups. However, LDL levels at the time of the gout diagnosis were significantly higher in the hepatotoxicity group than those in the no-hepatotoxicity group (142.0 [119.0–165.0] vs. 108.0 [82.0–129.0], *p* = 0.01).

### 3.3. Logistic Regression Analysis for Hepatotoxicity in Gout Patients on Febuxostat

Univariate logistic regression analysis revealed that pre-existing liver disease was significantly associated with an increased risk of hepatotoxicity (odds ration [OR], 4.046; 95% confidence interval [CI], 1.439–11.375; *p* = 0.008). After adjusting for age, febuxostat dosage, ALT, and hyperlipidemia, underlying liver disease was independently associated with a 4.1-fold increase in the risk of developing hepatotoxicity (OR, 4.083; 95% CI, 1.326–12.577; *p* = 0.014) (Table 3). A subgroup analysis excluding liver cirrhosis revealed that fatty liver was also an independent risk factor for the development of hepatotoxicity after febuxostat usage (OR, 2.353; 95% CI, 1.320–4.197; *p* = 0.004).

### 3.4. Side Effects of Colchicine

Thirteen (10.7%) of 121 patients treated with colchicine and febuxostat had acute liver injury, two (1.6%) patients had diarrhea, and one (0.8%) patient had a skin rash within three months after colchicine treatment. Meanwhile, of the 57 patients treated with febuxostat, four (7.0%) presented with only an acute liver injury [7.0% (febuxostat monotherapy group) vs. 10.7% (colchicine and febuxostat combination therapy group), *p* = 0.587] and two presented with diarrhea [3.5% (febuxostat monotherapy group) vs. 1.6% (colchicine and febuxostat combination therapy group), *p* = 0.594]; no patient developed a skin rash (0% vs. 0.8%, *p* = 1.0). There were no patients with muscle pain, muscle weakness, or neurotoxicity. Of the 13 patients who developed hepatotoxicity, nine continued to receive colchicine treatment, while four discontinued it. Ten patients (76.9%) used hepatotonics. In all patients with hepatotoxicity, liver function parameters recovered to their normal ranges or remained stable compared to their previous levels.

## 4. Discussion

In the present study, prophylactic colchicine did not increase the risk of acute hepatotoxicity in gout patients on febuxostat. However, in these patients, pre-existing liver disease may be associated with an increased risk of hepatotoxicity.

Gout is a common chronic inflammatory arthritis [1]. Recently, the incidence of younger gout patients has been increasing faster than older patients [2,11]. Therefore, gout is considered an important public healthcare issue. The goal of long-term treatments of gout is to reduce the levels of serum urate, subsequently avoiding acute gout attacks and inhibiting progression to chronic arthropathy. A uric acid-lowering agent is effective for lowering serum urate levels, and reduces the rate of gout flares and tophus burden [12]. However, during the initial use of ULT, rapid reduction in serum uric acid levels can often cause flares of gout, especially in the situation of in-patients, diuretics use, surgery, and overhydration [13,14,15]. Acute gout flare is a clinically evident episode of articular or periarticular inflammation induced by monosodium urate crystals [16], causing severe pain and disability of the articular joint. Therefore, gout flare is one of the most important concerns for patients as it can also affect their quality of life [17,18]. The European League Against Rheumatism (EULAR) recommendations have suggested that anti-inflammatory agents, such as low-dose colchicine or nonsteroidal anti-inflammatory drugs, should be used for at least six months when initiating ULT [6]. Previous studies reported that prophylactic treatment longer than six months is associated with fewer gout flares after initiating ULT [19,20].

Colchicine is an anti-inflammatory agent that has long been used to relieve pain and inflammation in acute gout attacks [21]. It inhibits the release of crystal-induced chemotactic factors from neutrophil lysosomes, blocks neutrophil adhesion to the endothelium, and reduces monosodium urate crystal-induced production of superoxide anions from neutrophils [22,23]. Therefore, colchicine effectively controls and prevents acute gout flare. However, it has also several toxicities, including gastrointestinal, renal, neuromuscular, hepatic, and cerebral toxicity, and bone marrow suppression [24,25,26].

When initiating colchicine in patients with gout, it is necessary to carefully check their comorbidities, and concomitant medications. There is a controversy around hepatotoxicity after colchicine treatment. Experimental studies have shown that colchicine causes hepatotoxicity, including acute hepatic necrosis and steatosis in animals [9,27]. Guo X. et al. reported that CYP3A inhibition was associated with colchicine-induced hepatotoxicity in animals [28]. However, a meta-analysis study demonstrated that adverse liver events did not increase in gout patients with colchicine use [29]. The present study also revealed that the number of patients with hepatotoxicity was not significantly higher in colchicine users than non-users. In addition, colchicine in patients with febuxostat did not increase their other side effects. Based on a previous meta-analysis and the present study results, colchicine can be safely used to prevent acute flares in gout patients on febuxostat.

Recent studies have shown that gout and hyperuricemia are significantly associated with metabolic syndrome [4]. Especially, hepatic steatosis and non-alcoholic fatty liver disease in younger gout patients have increased due to prevalent obesity and western dietary habits. Therefore, when treating hyperuricemia, hepatotoxicity has caused problems in these patients. A previous report demonstrated that febuxostat is associated with low risk of hepatotoxicity in Korean gout patients [8]. However, a recent randomized-controlled study from Huang et al. revealed that liver function abnormality was the most common adverse side-effect in gout patients treated with 80mg of febuxostat; febuxostat was discontinued in about 10% of the patients due to liver dysfunction [30]. Therefore, when initiating febuxostat therapy in patients with gout, it is important to identify the risk factors for development of hepatotoxicity in these patients. Our study demonstrated that febuxostat can increase the liver enzyme levels in patients with underlying liver diseases (such as fatty liver or liver cirrhosis). Therefore, we suggest the careful monitoring of liver function tests in patients with underlying liver disease after initiation of ULT.

There are several limitations to this study. First, the present study is a retrospective cohort design and the study populations were composed of a single medical center. Therefore, the number of study patients was relatively small and could introduce selection bias. Second, the liver diseases, including liver cirrhosis and fatty liver, were not confirmed by liver biopsy but rather diagnosed by imaging studies. Third, since hepatic side effects were defined by laboratory results, we could not exclude other causes of hepatotoxicity. Finally, it is possible that the adverse events of colchicine and gout flares may have been underestimated due to the retrospective design.

## 5. Conclusions

In conclusion, colchicine as a prophylactic therapy was not associated with acute hepatotoxicity in gout patients initiating febuxostat. Therefore, colchicine can be safely combined with febuxostat in gout patients without fatty liver or liver cirrhosis. However, attention needs to be paid to use of febuxostat in patients with pre-existing liver diseases.

## Figures and Tables

**Table 1:** Comparison of baseline characteristics according to the use of colchicine.

	Colchicine User (*N* = 121)	Colchicine No-User (*N* = 57)	*p* Value
Age, years	51.0 (37.0–62.0)	56.0 (43.5–68.5)	0.203
Male	119 (98.3)	49 (86.0)	0.002
Disease duration, months	26.6 (15.4–61.7)	23.9 (16.4–41.6)	0.748
Symptom duration, months	36.1 (5.2–73.3)	13.6 (0.8–55.2)	0.134
Duration of febuxostat use, months	17.6 (10.3–27.7)	20.8 (14.5–31.0)	0.165
Dosage of febuxostat, mg/day	59.2 ± 21.5	56.8 ± 19.9	0.491
Duration of colchicine use, months	13.3 (6.9–21.5)		
Dosage of colchicine, mg/day	0.6 ± 0.2		
Presence of tophi	24 (19.8)	9 (15.8)	0.680
Renal stone	9 (7.4)	5 (8.8)	1.0
Family history	9 (7.4)	4 (7.0)	1.0
Previous history of cancer	8 (6.6)	4 (7.0)	1.0
Gout flares within 3 months	14/113 (12.4)	24/51 (47.1)	<0.001
**Comorbidities**			
Hypertension	46 (38.0)	34 (59.6)	0.010
Diabetes mellitus	10 (8.3)	14 (24.6)	0.005
Cerebrovascular disease	15 (12.4)	14 (24.6)	0.051
Heart failure	1 (0.8)	4 (7.0)	0.037
Dyslipidemia	31 (25.6)	14 (24.6)	1.0
Hypertriglyceridemia	56 (46.3)	23 (40.4)	0.589
Liver disease	24 (19.8)	13 (22.8)	0.694
Chronic kidney disease (eGFR < 60 mL/min/1.73 m^2^)	8 (6.6)	19 (33.3)	<0.001
Dementia	1 (0.8)	1 (1.8)	0.539
**Laboratory findings**			
Uric acid (mg/dL)	8.6 (7.0–9.9)	8.4 (6.9–9.8)	0.618
AST (IU/L)	29.0 (23.0–36.0)	26.5 (23.0–37.8)	0.163
ALT (IU/L)	31.5 (22.0–44.3)	27.5 (19.0–43.0)	0.465
BUN (mg/dL)	15.3 (12.9–19.3)	18.9 (13.3–27.1)	0.139
Cr (mg/dL)	1.0 (0.8–1.1)	1.1 (0.9–1.8)	0.006
eGFR (mL/min/1.73 m^2^)	89.0 (75.0–104.5)	70.0 (34.0–96.5)	0.020
Total cholesterol (mg/dL)	190.0 (154.0–216.0)	171.5 (146.5–203.8)	0.078
Triglyceride (mg/dL)	207.0 (123.0–292.0)	199.5 (129.0–260.5)	0.897
LDL (mg/dL)	112.5 (86.3–135.0)	106.0 (82.0–128.0)	0.268
HDL (mg/dL)	46.0 (40.0–51.0)	43.0 (37.3–54.0)	0.508
**Medications**			
Aspirin	14 (11.6)	13 (22.8)	0.072
Diuretics	8 (6.6)	15 (26.3)	0.001

Results are expressed as the mean ± SD, as the median (interquartile range, IQR), or as number (%). AST, aspartate aminotransferase; ALT, alanine aminotransferase; BUN, blood urea nitrogen; Cr, creatinine; eGFR, estimated glomerular filtration rate; LDL, low-density lipoprotein; HDL, high-density lipoprotein.

**Table 2:** Comparison of baseline characteristics according to hepatotoxicity in gout patients with febuxostat.

	Hepatotoxicity (*N* = 17)	No Hepatotoxicity (*N* = 161)	*p* Value
Age, years	38.0 (34.0–60.0)	54.0 (39.0–64.0)	0.166
Male	17 (100.0)	151 (93.8)	0.601
Disease duration, months	24.3 (17.8–91.1)	26.1 (15.4–47.5)	1.0
Symptom duration, months	18.2 (0.8–90.2)	25.1 (3.9–67.9)	0.793
Duration of febuxostat use, months	17.5 (6.6–27.0)	19.2 (11.7–29.0)	0.645
Dosage of febuxostat, mg/day	50.6 ± 20.1	59.3 ± 21.0	0.109
Use of colchicine	13 (76.5)	108 (67.1)	0.587
Duration of colchicine use, months	7.0 (3.9–25.3)	13.4 (7.4–21.5)	0.975
Dosage of colchicine, mg/day	0.6 ± 0.2	0.6 ± 0.2	0.858
Presence of tophi	3 (17.6)	30 (18.6)	1.0
Gout flares	2 (11.8)	39 (24.2)	0.365
**Comorbidities**			
Hypertension	6 (35.3)	74 (46.0)	0.452
Diabetes mellitus	3 (17.6)	21 (13.0)	0.706
Cerebrovascular disease	2 (11.8)	27 (16.8)	1.0
Heart failure	1 (5.9)	4 (2.5)	0.398
Dyslipidemia	7 (41.2)	38 (23.6)	0.142
Hypertriglyceridemia	7 (41.2)	70 (43.4)	1.0
Liver disease	8 (47.1)	29 (18.0)	0.010
Chronic kidney disease (eGFR < 60 mL/min/1.73 m^2^)	1 (5.9)	26 (16.1)	0.476
Dementia	1 (5.9)	1 (0.6)	0.182
**Laboratory findings**			
Uric acid (mg/dL)	8.6 (7.0–9.8)	8.5 (6.9–9.8)	1.0
AST (IU/L)	30.0 (26.5–44.8)	28.0 (23.0–35.3)	0.402
ALT (IU/L)	42.5 (20.0–76.3)	29.0 (21.0–41.3)	0.755
BUN (mg/dL)	13.3 (10.1–19.3)	15.9 (13.2–22.4)	0.793
Cr (mg/dL)	1.0 (0.8–1.2)	1.0 (0.9–1.2)	0.925
eGFR (mL/min/1.73 m^2^)	84.0 (78.3–103.0)	85.5 (63.8–104.0)	0.8
Total cholesterol (mg/dL)	195.0 (157.0–230.0)	176.5 (151.0–210.3)	0.1
Triglyceride (mg/dL)	215.5 (137.8–282.8)	199.0 (123.0–292.0)	0.784
LDL (mg/dL)	142.0 (119.0–165.0)	108.0 (82.0–129.0)	0.01
HDL (mg/dL)	44.0 (40.0–51.0)	46.0 (39.0–52.0)	0.982
**Medications**			
Aspirin	3 (17.6)	24 (14.9)	0.726
Diuretics	1 (5.9)	22 (13.7)	0.702

Results are expressed as the mean ± SD, as the median (interquartile range, IQR), or as number (%). AST, aspartate aminotransferase; ALT, alanine aminotransferase; BUN, blood urea nitrogen; Cr, creatinine; eGFR, estimated glomerular filtration rate; LDL, low-density lipoprotein; HDL, high-density lipoprotein.

**Table 3:** Risk factors for hepatotoxicity in gout patients on febuxostat.

Baseline Variables	Univariate		Multivariate	
	OR (95% CI)	*p*	OR (95% CI)	*p*
Age	0.975 (0.946–1.006)	0.114	0.976 (0.941–1.013)	0.198
Duration of febuxostat use	0.990 (0.956–1.025)	0.573		
Febuxostat dosage	0.978 (0.952–1.005)	0.113	0.976 (0.946–1.006)	0.120
Colchicine use	1.595 (0.496–5.128)	0.433		
Duration of colchicine use	0.998 (0.954–1.043)	0.917		
Colchicine dosage	0.700 (0.015–32.928)	0.856		
ALT	1.016 (0.998–1.034)	0.082	1.010 (0.987–1.033)	0.415
LDL	1.0 (0.997–1.003)	0.821		
Hyperlipidemia	2.266 (0.807–6.360)	0.120	1.855 (0.581–5.920)	0.296
Chronic kidney disease	0.325 (0.041–2.555)	0.285		
Liver disease	4.046 (1.439–11.375)	0.008	4.083 (1.326–12.577)	0.014

Adjusted for age, febuxostat dose, ALT, hyperlipidemia and liver disease. OR, odds ratio; CI, confidence interval; ALT, alanine aminotransferase; LDL, low-density lipoprotein.
